# Simple cubic self-assembly of PbS quantum dots by finely controlled ligand removal through gel permeation chromatography†

**DOI:** 10.1039/d1sc02096j

**Published:** 2021-07-05

**Authors:** Jianjun Liu, Kazushi Enomoto, Kotaro Takeda, Daishi Inoue, Yong-Jin Pu

**Affiliations:** RIKEN Center for Emergent Matter Science (CEMS) Wako Saitama 351-0198 Japan yongjin.pu@riken.jp

## Abstract

The geometry in self-assembled superlattices of colloidal quantum dots (QDs) strongly affects their optoelectronic properties and is thus of critical importance for applications in optoelectronic devices. Here, we achieve the selective control of the geometry of colloidal quasi-spherical PbS QDs in highly-ordered two and three dimensional superlattices: Disordered, simple cubic (sc), and face-centered cubic (fcc). Gel permeation chromatography (GPC), not based on size-exclusion effects, is developed to quantitatively and continuously control the ligand coverage of PbS QDs. The obtained QDs can retain their high stability and photoluminescence on account of the chemically soft removal of the ligands by GPC. With increasing ligand coverage, the geometry of the self-assembled superlattices by solution-casting of the GPC-processed PbS QDs changed from disordered, sc to fcc because of the finely controlled ligand coverage and anisotropy on QD surfaces. Importantly, the highly-ordered sc supercrystal usually displays unique superfluorescence and is expected to show high charge transporting properties, but it has not yet been achieved for colloidal quasi-spherical QDs. It is firstly accessible by fine-tuning the QD ligand density using the GPC method here. This selective formation of different geometric superlattices based on GPC promises applications of such colloidal quasi-spherical QDs in high-performance optoelectronic devices.

## Abstract 2

Gel permeation chromatography can finely control ligand coverage of PbS quantum dots. Self-assembly of these QDs with different ligand density leads to the formation of 2D square, hexagonal and 3D simple cubic and face-centered cubic superlattices.

## Graphical abstract

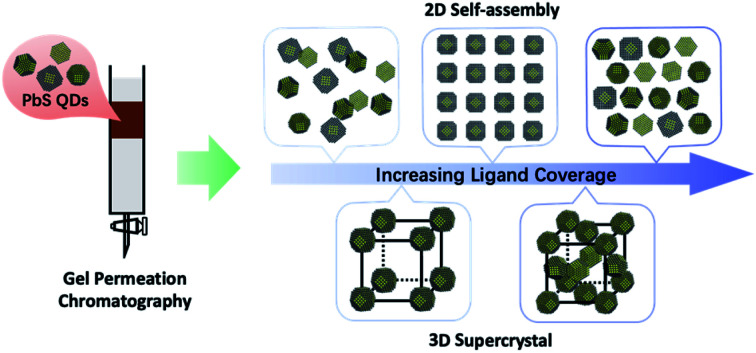

## Introduction

Colloidal quantum dots (QDs) have attracted substantial attention due to their characteristic optoelectronic properties based on their size confinement effects.^1,2^ They are also known to form highly ordered superlattices in the self-assembled solid state.^3^ The geometry of such self-assembled QDs has been explored theoretically^4,5^ and experimentally in order to better understand the ensemble effects on their optical and electrical properties, especially with regard to solid-state device applications.^6–13^ Accordingly, it is essential to selectively prepare two- (2D) and three-dimensional (3D) QD superlattices with different geometry and crystallographic patterns.

As far as 2D QD superlattices are concerned, QD monolayers with square or hexagonal arrangements are easily prepared by liquid/air interface methods with ligand-removal reagents,^14–18^ and their different band structures and charge-transporting properties have been well documented.^6–8,19^ As far as 3D QD superlattices are concerned, the so-called QD supercrystals, QDs with a shape close to spherical readily self-assemble into close-packed structures, *i.e.*, face-centered cubic (fcc) or hexagonal close packing (hcp), with a maximum packing fraction of 0.74.^20–22^ Body-centered cubic (bcc) structures with a slightly lower packing fraction of 0.68 can also be obtained by modification of the QD ligand coverage.^23,24^ However, 3D QD supercrystals with a simple cubic (sc) structure, which theoretically exhibit high charge transport properties compared to other 3D self-assembled superlattices according to first-principles calculations,^4^ are difficult to obtained from sphere-like (non-cubic) QDs because of a relatively low packing fraction of 0.52 and the low stability by entropy.^25^ Such sc superlattices have been reported predominantly for cubic-shaped QDs such as CsPbBr_3_ perovskite QDs due to their intrinsically higher packing fraction.^5,26,27^ There are few reports on multilayer QD thin films that exhibit an sc arrangement of epitaxially fused quasi-spherical QDs at the liquid/air interface with ethylenediamine as the ligand remover.^18^ Therefore, 3D supercrystals of self-assembled quasi-spherical QDs with an sc arrangement remain a superbly challenging research target that has, to the best of our knowledge, not yet been achieved.

Precipitation in a poor solvent and redispersion in a good solvent (PR),^20^ the liquid/air interface with a ligand-removing-reagent method,^28^ and oxidative removal in air^21^ are effective options to modify the ligand coverage and thereby control the QD self-assembly geometry based on anisotropic ligand distribution on the QD surface. However, such techniques to control the ligand coverage render the solubility, processability, and stability of the QDs poor because controlling the degree of ligand removal on the QDs is difficult on account of their high reactivity. Currently, the liquid/air interface method is widely used to form ‘confined-but-connected’ epitaxially fused QD superlattice structures by excessive ligand removal,^7,8,19,29^ resulting in a severe deterioration in the photoluminescence quantum yield (PLQY),^30^ and the resulting QD self-assembled superlattices are thus difficult to use in luminescence-related applications.

Here, we develop gel permeation chromatography (GPC) as a method to remove ligands from PbS QDs in solution. GPC with a size-exclusion effect for the purification and separation of colloidal QDs has been reported to remove residual long alkyl ligands and high-boiling solvents such as octadecene after synthesis.^31–35^ On the other hand, the GPC method in this study controls ligand coverage of the QDs and does not show any size-exclusion effect on the size of the QDs. This GPC process enables the soft, quantitative, and continuous control over the ligand coverage of the QDs, which retain sufficient solubility for processability and luminescence properties. The different self-assembled superlattices of GPC-processed QDs can be prepared predictively and selectively, not only with 2D (square and hexagonal) arrangements, but also with 3D (sc and fcc) arrangements. It is worth noting that unusual highly long-range-ordered 2D square arrays and 3D sc supercrystals along the [100]_QD_ facet can be prepared from quasi-spherical PbS QDs by direct solution-casting on solid substrates. At this point, we think that the mechanism for the selective formation of different self-assembly geometries is due to the anisotropy on the QD surface as a result of the gentle ligand removal by GPC.

## Results

### GPC process and control over the ligand density

Colloidal PbS QDs with oleic acid (OA) ligands were synthesized from lead chloride (PbCl_2_) according to a literature procedure.^36^ After the synthesis, a QD sample was purified once by PR prior to the GPC process, as unreacted precursors should be removed while as many ligands as possible should remain on the QD surface; this sample was named before-GPC. Transmission electron microscopy (TEM) measurements of the before-GPC sample revealed uniform sphere-like structures with an average diameter of 7.3 nm (Fig. S1a and S3a†). X-ray diffraction peaks confirmed a rock salt structure typical for PbS bulk (Fig. S1b†).

**Fig. 1:**
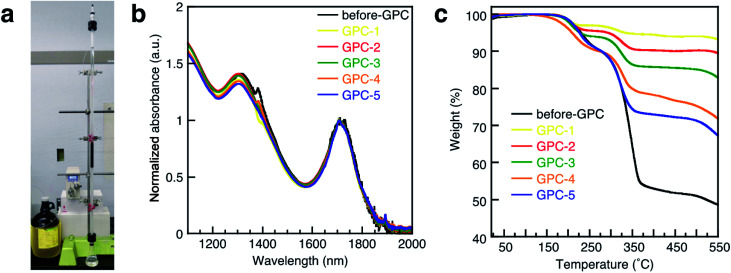
(a) Photograph of the GPC system used in this study. (b) Absorption spectra of PbS QDs in tetrachloroethylene (TCE) solution for before-GPC and GPC1-5. (c) TGA curves of PbS QDs for before-GPC and GPC1-5.

For the GPC process (Fig. 1a), 10.0 g of polystyrene beads (stationary phase) were filled in a glass column (diameter: 1.0 cm; effective column length: 72 cm; eluent: toluene). After injecting the QD solution (2.0 mL), the flow rate of the eluent was set to 0.8 mL min^−1^. The eluted QD solution was collected in five consecutive portions of 0.8 mL (GPC-1 to GPC-5). The absorption spectra for before-GPC and GPC-1–5 in tetrachloroethylene (Fig. 1b) were identical and peak shifts were not observed, suggesting that the size of all QDs in all the samples is identical, excluding the possibility of fusion and/or coupling between the QDs during the process.^37^ TEM measurements also confirmed the identical QD size in all samples (Fig. 3a and S7a†). These results clearly demonstrate that the GPC process does not exert any size-exclusion effect on the size of the QDs and that undesirable side reactions do not occur.

**Fig. 2:**
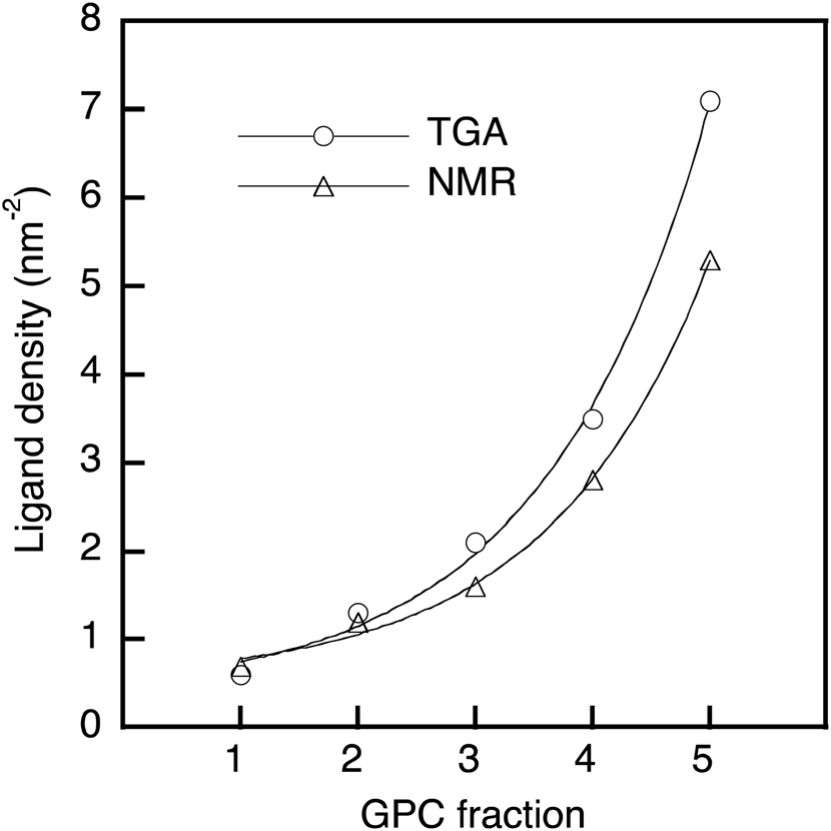
Ligand density of PbS QDs as a function of the GPC retention time; solid line: exponential fitting.

A thermogravimetric analysis (TGA) was carried out in order to evaluate the ligand coverage of the PbS QDs (Fig. 1c). In the TGA curves of all samples, there are two obvious weight-loss events at approximately 220 °C and 330 °C, which correspond to the decomposition of free OA and bound OA, respectively (Fig. S2†).^38^ Based on these two different weight-loss events, we calculated that before-GPC QDs contain 10.4 wt% of free OA and 35.9 wt% of bound OA. Among the GPC-processed QDs, the weight loss for bound OA gradually increased from GPC-1 (2.6 wt%) to GPC-5 (22.5 wt%). These weight losses of bound OA can be converted into weight ratios of OA ligands bound to a QD. In order to estimate the number of ligands on a QD, *i.e.*, in order to transform the weight ratio into a ligand density, the atomic Pb/S ratio of the QDs is required. For that purpose, we used Rutherford backscattering (RBS) spectrometry (Fig. S3†) of the before-GPC and GPC-2 samples, which revealed Pb/S ratios of 1.29 (before-GPC) and 1.25 (GPC-2). These values are consistent with that reported by Moreels *et al.* for PbS QDs synthesized from PbCl_2_ (1.26).^39^ The average Pb/S ration (1.27) was subsequently used for the estimation of the ligand density (GPC-1: 0.6 nm^−2^; GPC-2: 1.3 nm^−2^; GPC-3: 2.1 nm^−2^; GPC-4: 3.5 nm^−2^; GPC-5: 7.1 nm^−2^) (Table 1 and Fig. 2). It should be noted here that the value for GPC-5 is most likely an overestimation given that it is higher than the theoretically expected maximum.^40^

**Table 1:** Ligand density of PbS QDs in GPC1-5 estimated by TGA and NMR

Sample	TGA			NMR			
	Ligand/QD (wt/wt)	Ligand/QD	Ligand density (nm^−2^)	Ligand concn. (μmol mL^−1^)	QD concn. (μmol mL^−1^)	Ligand/QD	Ligand density (nm^−2^)
GPC-1	0.028	99	0.6	0.316	0.0226	121	0.7
GPC-2	0.059	211	1.3	0.737	0.0237	198	1.2
GPC-3	0.098	352	2.1	0.936	0.0360	260	1.6
GPC-4	0.163	586	3.5	1.649	0.0348	474	2.8
GPC-5	0.332	1190	7.1	3.088	0.0347	891	5.3

We also estimated the ligand density by ^1^H nuclear magnetic resonance (NMR) spectroscopy using ferrocene as an internal standard. The NMR spectra showed two peaks corresponding to vinyl protons of the free and bound OA ligands at 5.45 ppm and 5.60 ppm, respectively (Fig. S4†).^32,41^ The peak intensity of the bound OA was calibrated by subtracting the overlapping free OA peak, and the molar concentration of the bound OA in the NMR measurement was obtained *via* the internal standard.^32^ The molar concentration of the QDs was estimated using the empirical equation of Moreels *et al.*^39^ The obtained results for the ligand density (GPC-1: 0.7 nm^−2^; GPC-2: 1.2 nm^−2^; GPC-3: 1.6 nm^−2^; GPC-4: 2.8 nm^−2^; GPC-5: 5.3 nm^−2^) (Table 1 and Fig. 2) are consistent with the ligand density obtained from the TGA measurements.

These TGA and NMR measurements clearly demonstrate that the ligand density of the PbS QDs gradually increases from GPC-1 to GPC-5, and that the GPC method is thus able to continuously and precisely control the ligand density of the PbS QDs by collecting different fractions according to the retention time. This is far superior to other methods of ligand-coverage control such as the PR method, the liquid/air interface method, and oxidatively removing ligands in air. Another advantage of the GPC method for controlling the ligand coverage of colloidal QDs is that the QD samples retain their solubility in toluene and are stable in solution during and after the GPC process.

In this GPC process, the QDs with fewer ligands elute early, while the QDs with more ligands elute later. Interestingly, this order stands in sharp contrast to conventional GPC methods, given that QDs with fewer ligands should have a smaller hydrodynamic volume in toluene than those that contain more ligands. Therefore, ligand control by the GPC method in this study is not based on a size-exclusion effect, which is further corroborated by the exponential increase of the ligand density from GPC-1 to GPC-5 (Fig. 2). At this point, we postulate that the bound ligands are gradually removed during the GPC process even in non-polar toluene, probably by trace amounts of residual polar water. To investigate the effect of water in toluene, we used super anhydrous toluene instead of normal-grade toluene as the eluent in the GPC under otherwise identical conditions. The TGA curves of these GPC samples (Fig. S5†) showed that the weight loss corresponding to the ligands gradually decreased from GPC-5 to GPC-1, albeit that the difference (6.6%) is substantially smaller than that of the GPC samples eluted with normal-grade toluene (19.9%). This result suggests that the solvent purity for the GPC method affects the removal of the ligands from the QDs, and that the ligands are chemically detached. Nevertheless, we still do not fully understand why the retention time in the GPC causes the increase of the ligand density. It may be because the ligands that are removed from early-eluting QD samples remain in the column to gradually retard removal of ligands from later-eluting QD samples by shifting ligand binding/detaching equilibria in the solvent. Also, QDs with fewer ligands would exhibit a more polar surface than QDs with more ligands, and less interactions with the hydrophobic stationary phase would make QDs with fewer ligands elute early.

### Self-assembled 2D superlattices

**Fig. 3:**
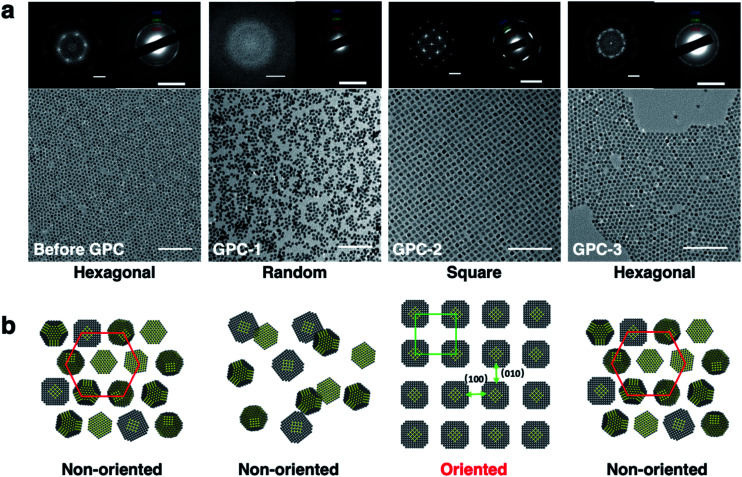
(a) TEM images of the 2D self-assemblies obtained for before-GPC, GPC-1, GPC-2, and GPC-3 QDs; inset: FFT and SAED patterns; scale bars: 100 nm (TEM), 0.1 nm^−1^ (FFT), and 5 nm^−1^ (SAED). (b) Schematic diagrams of the corresponding 2D self-assemblies.

The 2D self-assembly of the PbS QDs was achieved by drop-casting dilute toluene solutions onto solid substrates. Before-GPC and GPC-1–5 formed 2D superlattices with different and unique geometric arrangements that were examined by TEM (Fig. 3a). Before-GPC QDs formed a long-range-ordered hexagonal lattice, which is common for quasi-spherical PbS QDs. However, GPC-1, GPC-2, and GPC-3 QDs adopted different 2D self-assembled geometries: random, square, and hexagonal, respectively. GPC-1 QDs showed a random assembly without the formation of a superlattice, probably due to insufficient ligands on the QD surface. It is noteworthy that GPC-2 QDs formed a self-assembled 2D superlattice with a square geometry, wherein no contact and/or fusion between QDs was observed. This result stands in contrast to previous reports on fused square 2D superlattices, which required post-treatment ligand removal.^6–9^ A selected area electron diffraction (SAED) analysis of this square superlattice revealed a clear diffraction pattern, which was ascribed to the [200] facet of the PbS crystals, along the direction of the square arrangement, demonstrating that the GPC-2 QDs in the square superlattice are highly oriented along the [100]_QD_ facets (Fig. 3b). Importantly, the 2D square lattice of GPC-2 QDs extends over several micrometers with high uniformity, which is reflected in sharp SAED peaks (Fig. S6†). The GPC-3–5 QDs formed hexagonal 2D assemblies with non-oriented crystal facets (Fig. S7†), similar to the before-GPC QDs. The average center-to-center distance between the QDs was estimated based on Fast Fourier Transform (FFT) pattern of the TEM images (Fig. 3a). The distance between GPC-2 QDs along the [100]_QD_ direction in the square superlattice (12.2 nm) is longer than that in the hexagonal lattices of the before-GPC and GPC 3–5 QDs (9.2–9.6 nm). This difference may be attributed to the different interparticle interactions between QDs in square and hexagonal assembled superlattices. The direct weak [100]_QD_-to-[100]_QD_ facets interactions between GPC-2 QDs in oriented square superlattices can produce a longer interparticle spacing. In contrast, the different direct facet-to-facet contacts ([100]-to-[100]; [100]-to-[111]; [111]-to-[111]), especially the strong [111]-to-[111] contacts, and the orientation of ligands at [111]_QD_ can strengthen the packing density of surface ligands and QD–QD interactions to reduce the interparticle spacing in other hexagonal superlattices.^22^

### Self-assembled 3D supercrystals

**Fig. 4:**
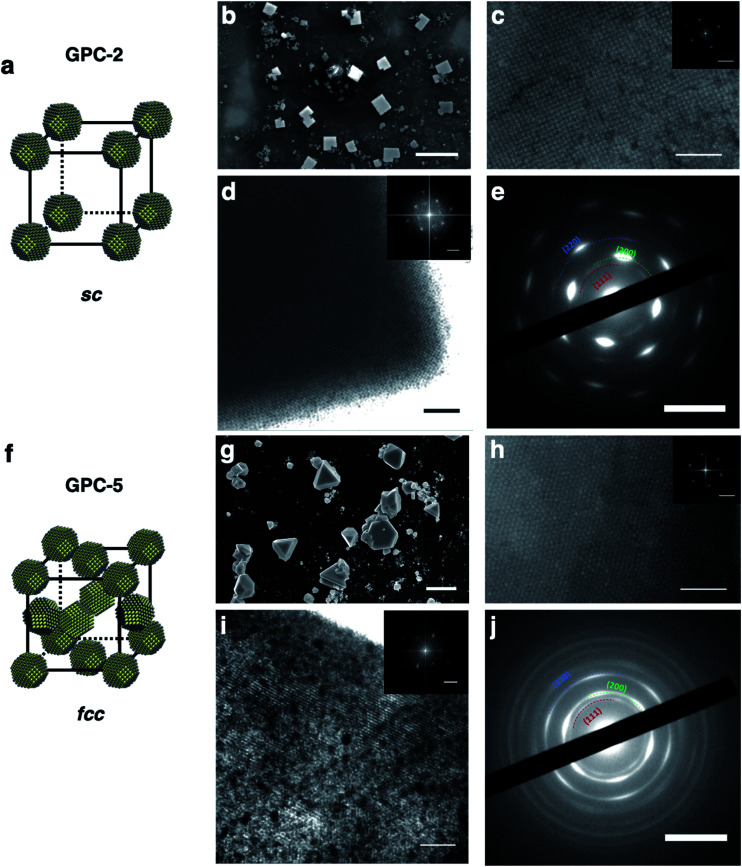
3D self-assembly supercrystals of GPC-2 and GPC-5 QDs. (a) and (f) schematic model of the sc and fcc superlattices. (b) and (g) SEM images of the supercrystals. (c) and (h) HR-SEM images of the supercrystal surfaces; inset: FFT pattern. (d) and (i) TEM images of the supercrystals along the edges; inset: FFT pattern. (e) and (j) SAED patterns of the supercrystals; scale bars: (b) and (g) 10 μm (SEM of the crystals); (c) and (h) 100 nm (SEM of the crystal surface) and 0.1 nm^−1^ (FFT); (d) and (i) 100 nm (TEM) and 0.1 nm^−1^ (FFT); (e) and (j) 5 nm^−1^ (SAED).

Based on the different 2D self-assembly geometries of the GPC samples, GPC-2 and GPC-5 were used as representative PbS QD samples for self-assembly into long-range-ordered 3D supercrystals. GPC-5 QDs formed triangular or hexagonal supercrystals (Fig. 4g and S8b†), which are commonly obtained for large (>5 nm) quasi-spherical QDs covered with OA ligands.^42^ The triangular shape of the supercrystals suggests that they are formed on the [111]_supercrystal_ plane of the fcc packing from a hexagonal layer on the substrate.^43^ High-resolution scanning electron microscopy (HR-SEM), TEM, and scanning transmission electron microscopy (STEM) measurements of the supercrystals supported this fcc supercrystal structure of the GPC-5 QDs (Fig. 4h, i and S9b†). The supercrystal of the GPC-5 QDs exhibits a hexagonal arrangement on the crystal surface that corresponds to the [111]_supercrystal_ facets of the fcc packing, which is consistent with previously reported fcc 3D supercrystals.^22^ An FFT analysis of the images (inset of Fig. 4h and i) revealed that the interparticle distance in the 3D supercrystals (8.2 nm) is shorter than that in the corresponding 2D superlattice (9.5 nm). This result is consistent with previously reported results, and partly attributed to potential ligand removal during the 3D self-assembly^44,45^ and/or tighter packing between QDs in the 3D supercrystal compared with the 2D monolayer. Unlike the 2D hexagonal superlattices with non-oriented crystal facets for GPC-5 QDs, the 3D fcc supercrystal exhibited broad hexagonal electron diffraction peaks ascribed to the internal crystal structure of the QDs in the SAED measurements (Fig. 4j). This result suggests that the PbS QDs in the fcc supercrystals are highly aligned along their [111]_QD_ facets in the [111]_supercrystal_ direction, which is consistent with previously reported fcc PbS QD supercrystals.^20,22^

GPC-2 QDs self-assembled in cubic supercrystals with sc packing (Fig. 4b and S8a†), which is usually difficult for quasi-spherical QDs on account of the low maximum-packing fraction (0.52). To the best of our knowledge, this is the first example of an sc supercrystal from quasi-spherical colloidal PbS QDs. The cubic shape of the supercrystals suggests that the supercrystal grows uniformly in the *x*, *y*, and *z* directions, which results from the sc packing of the QDs. HR-SEM, TEM, and STEM images of the supercrystals (Fig. 4c, d and S9a†) showed a square QD arrangement. A TEM image at the edge of the supercrystals revealed features typical for square lattices on the surface and inside of the crystals (Fig. 4d). The interparticle distance between the QDs in the supercrystal (10.9 nm), which was estimated based on an FFT analysis (inset of Fig. 4c and d), is significantly longer than that of the fcc supercrystal of the GPC-5 QDs (8.2 nm). This result follows the same trend as in the 2D self-assembly lattices. The larger interparticle distance is also a feature of the sc lattices, which are generally larger than the QD distance in other supercrystal structures (fcc, hcp, bcc) for QDs with OA ligands.^46^ SAED measurements also showed strong diffraction peaks for the [200]_QD_ facets at square positions (Fig. 4e), demonstrating highly oriented [100]_QD_ facets in the [100]_supercrystal_ layer. This result is consistent with the 2D square superlattice of GPC-2 QDs. The 3D multilayer structure of GPC-2 QDs thus offers important information on the QD arrangement in sc supercrystals. We observed the self-assembled multilayer structure of GPC-2 QDs (Fig. S10†). Higher contrast in the image corresponds to areas of increased thickness, and a square QD arrangement was widely observed regardless of the number of layers.^47^ This result excludes the possibility of bcc packing in the supercrystals.

It is noted that bcc packing as a common PbS QD assembly structure cannot be observed in GPC samples. Because of its lower packing density compared to fcc lattice, the self-assembly of bcc superlattice usually requires the adjustment of suitable external environment according to the bcc-related literatures,^48,49^ for example, the proper QD concentration, solvent type, evaporation rate and ligand coverage. However, all GPC samples here maintain the same self-assembly conditions. These self-assembly conditions make QDs tend to form fcc packing instead of bcc packing by comparison. On the other hand, although the bcc superlattices can be prepared by decreasing the ligand coverage on QD surface,^20,21^ Winslow *et al.*^41^ investigated the importance of unbound ligands in QD superlattice formation and revealed that unbound ligands in QD solution are detrimental to bcc superlattice formation and lead to a transformation of bcc into fcc superlattices. Therefore, a few unbound OA ligands in all GPC samples here may probably prevent the bcc superlattice formation and result in only sc and fcc superlattice formation.

### Stability and photoluminescence (PL) properties of the GPC samples

**Fig. 5:**
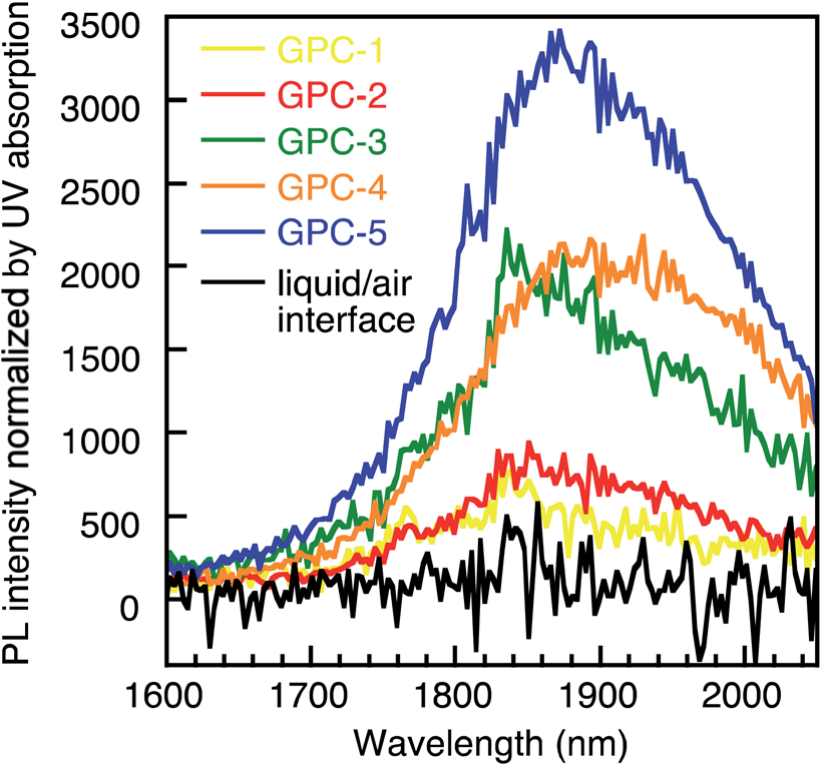
NIR-PL spectra of GPC-processed QDs in the solid state and for the liquid/air-interface QD film.

The all samples processed by the GPC method were soluble in toluene and exhibited good stability in the solution. Conversely, the PR and liquid/air interface methods always drastically remove ligands from the QD surface, which decreases the stability of the QDs. After three PR cycles (PR-3), the PbS QDs became insoluble and fused (Fig. S11†). Therefore, GPC exhibits unique advantages in soft ligand removal and modulation of QD self-assembled geometry which is difficult to reproduced by using other methods. We measured the PL spectra of films for the QDs processed by the GPC and liquid/air interface method (Fig. 5). All GPC QDs showed near-infrared (NIR) emission at approximately 1850 nm. The intensity normalized by the absorbance at the excitation wavelength (912 nm) gradually decreased from GPC-5 to GPC-1 due to quenching by increasing surface defects. However, QDs with fewer ligands, *e.g.* GPC-2, assembled in the sc superlattice, and still exhibited photoluminescence with reasonably high intensity. On the other hand, we also prepared a QD film from before-GPC by the liquid/air interface method (Fig. S12†).^6^ This film exhibited a 2D and 3D multilayer fused square superlattice in the TEM measurements, albeit that the uniform area was much narrower than in the square lattice from GPC-2 QDs. It should also be noted here that the QD film formed at the liquid/air interface showed almost no PL due to severe quenching during the assembly process. These results show that the GPC technique reported in this study allows not only tuning the self-assembly geometry of the QDs, but also keeps their stability in solution and ensures NIR emission in the solid state.

### Effect of the size of the QDs on the self-assembly geometry

To investigate the effect of the size of the QDs on the self-assembly after the GPC process, we synthesized smaller PbS QDs (diameter: 4.3 nm) with an absorption peak at 992 nm (Fig. S13†). Then, we applied the GPC method to these smaller QDs under conditions identical to those previously described. GPC-1–5 samples were collected and used for self-assembly experiments. After the GPC process, the small PbS QDs also showed tunable 2D self-assembly geometries with a random structure for GPC-1, a square structure for GPC-2, and hexagonal structures for GPC-3–5 (Fig. S13c†). GPC-2 QDs also assembled into cubic sc supercrystals (Fig. S13b†). Compared to the larger QDs (diameter: 7.3 nm), the small GPC-2 QDs formed a limited and narrower 2D square array area as well as smaller sc supercrystals (<0.5 micron). SAED measurements of the 2D square arrays and the 3D sc supercrystals obtained from the small PbS QDs showed amorphous ring patterns, indicating non-oriented QD facets. The shape of PbS QDs usually transforms from octahedral to truncated octahedral and cuboctahedral with increasing size from 1 nm to 8 nm. The [100] facets emerge at 4 nm, and the [100] facet area gradually widens and becomes clearer with increasing QD size.^50,51^ We presume that clear [100] facets cannot be formed on the surface of the small PbS QDs and that the crystallographic orientation is not achieved in the square and sc self-assembly superlattices.^52^

## Discussion

For the cuboctahedral QDs, previously reported theoretical calculations have shown that the ligand-binding energy on the [111] facet is higher than that on the [100] facet, and the relationship between the binding energy and the ligand density on the [111] and [100] facets has been examined by Bealing.^40^ Thus, although the distribution of ligands on different QD facets cannot be accurately evaluated by experiments, the ligand-removal process in our GPC method and the mechanism of their tunable self-assembly geometry can be discussed based on the ligand-density curve (Fig. 2) of GPC-processed QDs and the anisotropic character of the QD facets. In the GPC process, the QDs of GPC-3–5 elute later, which is commensurate with higher ligand density and leads to the formation of 2D hexagonal and 3D fcc self-assembly structures. This result was attributed to the adequate ligand distribution on the [111] and [100] facets and the small anisotropy of the facets, which causes the non-orientation of the crystals in the QD superlattices.^20^ On the other hand, in early-eluting GPC-2 QDs, commensurate with low ligand density, the ligands on the [100] facet are removed easier than the ligands on the [111] facet, and large anisotropic ligand distribution between [100] and [111] facets leads to a crystallographic orientation along the [100] facets in the 2D square and 3D sc self-assembly geometries. GPC-1 QDs self-assemble into random structures because the excessive ligand removal causes relatively low anisotropy. The GPC technique presented here can thus produce different degrees of anisotropy on PbS QD facets *via* the controllable ligand removal in solution, which leads to tunable multi-dimensional self-assembly geometries.

## Conclusions

We have reported a method to quantitatively control the ligand density of PbS quantum dots (QDs) using a gel permeation chromatography (GPC) technique. This GPC technique is, in contrast to conventional GPC methods, not based on a size-exclusion effect, but able to finely and continuously remove ligands coordinated to the surface of the PbS QDs, thus providing precise control over the ligand density in different GPC fractions. Direct solution-casting of colloidal PbS QDs with different ligand density led to the selective formation of 2D square and hexagonal superlattices as well as 3D supercrystals with simple cubic (sc) and face-centered cubic (fcc) packing. Based on the quantitatively controlled ligand coverage, the quasi-spherical PbS QDs can self-assemble into unusual square arrangements and sc supercrystals with highly ordered crystallographic orientation. Anisotropy on QD surfaces as a result of gentle ligand removal in the GPC is considered a reliable mechanism for the formation of distinct self-assembly geometries. The stability of the QDs in solution after controlling the ligand density and the near-infrared-photoluminescence (NIR-PL) in solid state are superior to those obtained using other ligand-removal techniques. It is obvious that this soft removal of ligands by GPC technique as a new function exhibits unique advantages in the modulation of QD self-assembled geometry and their optoelectronic properties. The various multidimensional self-assembly geometries of QDs by this operationally simple GPC method can thus be expected to provide significant insights into the formation mechanism and applications of QD superlattices. The significance of this GPC technique also inspires us to further investigate its potential applications to the functional metal or metal oxide nanoparticles with weakly bound ligands in the future.

## Author contributions

J. Liu synthesized and purified the QDs, and measured TGA, NMR, XRD, UV and PL. J. Liu and K. Enomoto measured TEM and SEM. K. Enomoto measured RBS. D. Inoue measured STEM. T. Kotaro contributed to get preliminary results on the GPC treatment of QDs. All experiments were performed under discussion with Y.-J. Pu. J. Liu, K. Enomoto, and Y.-J. Pu analyzed the data. J. Liu and Y.-J. Pu wrote the manuscript. All authors discussed and commented on the manuscript.

## Conflicts of interest

There are no conflicts to declare.

## Supplementary Material

SC-012-D1SC02096J-s001
