# Supplementary material for: Simple cubic self-assembly of PbS quantum dots by finely controlled ligand removal through gel permeation chromatography
Source: Chem Sci. 2021 Jul 5;12(30):10354–61. doi: 10.1039/d1sc02096j (PMC8336479; doi:10.1039/d1sc02096j)
Supplement: SC-012-D1SC02096J-s001 [file SC-012-D1SC02096J-s001.pdf]

Electronic Supplementary Material (ESI) for Chemical Science.
This journal is © The Royal Society of Chemistry 2021

Supporting Information

Simple Cubic Self-Assembly of PbS Quantum Dots by Finely Controlled Ligand Removal through Gel Permeation Chromatography

Jianjun Liu, [a] Kazushi Enomoto, [a] Kotaro Takeda, [a] Daishi Inoue [a] and Yong-Jin Pu *[a]

RIKEN Center for Emergent Matter Science (CEMS), Wako, Saitama, 351-0198, Japan

Email: yongjin.pu@riken.jp

1. Materials

1-Butanol (>99.0%), elemental sulfur powder (>98.0%), methanol (>99.8%), toluene (>99.5%), and toluene-d_8 (>99.5%) were purchased from Wako Pure Chemical Industries, Ltd., Osaka, Japan. *n*-Hexane (>96.0%) was purchased from Junsei Chemical Co., Ltd., Tokyo, Japan. Oleic acid (>90%, OA) was purchased from Sigma-Aldrich Co., LLC., Darmstadt, Germany. Ferrocene was purchased from TCI Ltd., Tokyo, Japan. Tetrachloroethylene (>99.0%, TCE) was purchased from Kanto Chemical CO. INC., Tokyo, Japan. All these chemicals were used as received.

Lead (II) chloride (Wako Pure Chemical Industries, Ltd., Osaka, Japan, >99.0%, $PbCl_2$) was dried *in vacuo* at 350 °C for 24 h and stored in a glove box. Oleylamine (Sigma-Aldrich Co., LLC., Darmstadt, Germany, >70%, OAm) was dehydrated *in vacuo* at 140 °C for 1 h and stored over activated molecular sieves 3Å 1/16 (Wako Pure Chemical Industries, Ltd., Osaka, Japan) in a glove box. Polystyrene divinylbenzene beads (S-X1, 200-400 mesh, Bio-Rad Laboratories, Inc., USA) were dried *in vacuo* at room temperature overnight and rinsed with toluene (300 mL) prior to use. Anhydrous toluene was obtained from a GlassContour solvent purification system, which was purchased from AS ONE Corporation, Osaka, Japan.

2. Synthesis of PbS QDs

PbS QDs (average diameter: 7.3 nm) were synthesized using the following, slightly modified literature procedure[1]: In a glove box, a four-neck flask (25 mL) was charged with elemental sulfur (0.040 g, 1.247 mmol) and OAm (7.5 mL). The mixture was heated at 120 °C for 5 min under nitrogen, before the temperature was decreased to 80 °C. Then, $PbCl_2$ (1.25 g, 3.5 mmol) and OAm (7.5 mL) were added into a separate four-neck flask (100 mL), and the mixture was heated at 140 °C for 30 min, which afforded a clear Pb-oleate solution. Then, the temperature was decreased to 110 ^{o}C and the sulfur solution (2.25 mL, 0.374 mmol) was injected into the Pb-oleate solution. After 30 min at 110 ^{o}C, the reaction was stopped by cooling in a water bath for 2 min, followed by injection of OA (20 mL) and further stirring for 2 min. An aliquot (5.0 mL) of the resulting dispersion was mixed with hexane (10 mL) and centrifuged at 4000 rpm for 3 min. The supernatant was further centrifuged at 4000 rpm for 3 min in order to remove any unreacted chemicals. Then, a butanol/methanol mixture (20 mL, 2:1, v/v) was added to the solution. The precipitated PbS QDs were collected by centrifugation at 4000 rpm for 3 min. The obtained powder was redispersed in toluene (1 mL) for the GPC measurements, and this sample is referred to as 'before-GPC' (PR1). Further precipitation and redissolution (PR) cycles (each cycle: 2 mL toluene and 4 mL butanol/methanol mixture; 4000 rpm/3 min) furnished PR2 (two cycles), PR3 (three cycles), and PR4 (four cycles).

Small PbS QDs (average diameter: 4.3 nm) were synthesized by a similar procedure. In a glove box, elemental sulfur (0.040 g, 1.247 mmol) and OAm (7.5 mL) were added to a four-neck flask (25 mL) before the mixture was heated at 120 °C for 5 min under nitrogen. Then, the temperature was decreased to 50 °C, before $PbCl_2$ (2.5 g, 7.0 mmol) and OAm (15 mL) were added into a separate four-neck flask (100 mL). The mixture was then heated at 140 °C for 30 min to afford a clear Pb-oleate solution. These reaction vessels were kept at their respective temperatures for 15 min, before the reaction mixture changed to a clear Pb-oleate solution. Then, the temperature was decreased to 50 ^{o}C and a sulfur solution (4.5 mL, 0.748 mmol) was injected

into the Pb-oleate solution. After 70 s, the reaction was stopped via the injection of OA (40 mL), followed by further stirring for 2 min. An aliquot (7.0 mL) of the resulting dispersion was mixed with toluene (10 mL) and centrifuged at 4000 rpm for 3 min. The supernatant was further centrifuged at 4000 rpm for 3 min (twice) in order to remove any unreacted chemicals. Then, a butanol/methanol mixture (20 mL, 2:1, v/v) was added to the obtained solution. The precipitated PbS QDs were collected by centrifugation at 4000 rpm for 3 min and the resulting powder was redispersed in toluene (2 mL) for GPC measurements.

3. Gel permeation chromatography (GPC)

GPC was conducted on polystyrene cross-linked with divinylbenzene as the stationary phase and toluene as the eluent. Polystyrene beads (10 g) were soaked in toluene (100 mL) overnight, before the beads in toluene were loaded into a glass column ($\Phi_{diameter}10*L_{length}1000$ mm). The flow rate of the eluent was set to 0.8 mL/min by peristaltic pumps to make the beads tightly packed, and the length of beads was stabilized at about 72 cm. Then, before-GPC (PR1) sample in toluene (2 mL) was carefully loaded onto the top of the column. When the sample liquid interface was level with the beads, toluene as an eluent was added to column and the peristaltic pumps was connected. The flow rate was set to 0.8 mL/min. When the QDs sample flowed through the GPC column and was just about to flow out from the bottom of the column, the GPC samples began to collect. To ensure the same volume of collected fractions, the time for collecting the samples was set to 1 min. When the QDs completely flowed out from the GPC column, the consecutive fractions of PbS QD solutions (0.8 mL) were collected and labeled in order of outflow as GPC-1 to GPC-5 for further experiments.

4. Self-assembly of 2D and 3D superlattices

2D self-assemblies of the GPC-processed QDs were prepared by slow solvent evaporation of the QD toluene solution (0.5 mg/mL) on a TEM grid under a toluene-saturated atmosphere. 3D self-assembly supercrystals were prepared by slow solvent evaporation of the QD toluene solution (5 mg/mL) on a TEM grid under a toluene-saturated atmosphere and by immersing silicon wafer into the QD toluene solution (5 mg/mL) follow slow solvent evaporation under a toluene-saturated atmosphere.

5. Measurements

TGA measurements were carried out on a TA-60 with a TGA-50 workstation (Shimadzu Co., Tokyo, Japan) at a heating rate of 10 °C/min under nitrogen. XRD measurements were carried out on a SmartLab (RIGAKU Co., Tokyo, Japan). PbS QDs samples in toluene (2.0 mg/mL) were drop-cast onto a reflection-free Si wafer, and dried *in vacuo* at room temperature. UV-Vis-NIR spectra were recorded on a UV-3600 Plus (Shimadzu Co., Tokyo, Japan) spectrophotometer. NIR-PL spectra were measured on a PMA-12 C10028-02 NIR detector (Hamamastu Co., Tokyo, Japan). Solution samples were prepared by dissolving QDs (0.5 mg) in TCE (3 mL). The film samples were obtained by drop-casting PbS QD toluene solutions (2 mg/mL) on quartz substrates, followed by drying *in vacuo* at room temperature. Microscopy and selected-area electron diffraction (SAED) images were measured using transmission electron microscopes operating at 200 kV (JEM-2100F/SP, JEOL Co., Tokyo, Japan) or 80 kV (JEM-1230, JEOL Co., Tokyo, Japan). Carbon-reinforced

polyvinyl formal membranes on Cu grids (Okenshoji Co., Tokyo, Japan, PVF-C10 STEM Cu100P grid) were used for the TEM measurements. Scanning electron microscopy (SEM) and STEM images were recorded on a Quattro S SEM (Talos, Thermo Fisher Scientific Co., Massachusetts, USA) operating at 5-30 kV. Samples for SEM measurements were prepared on TEM grids and hydrophilic Si wafers by self-assembly. NMR measurements were conducted on a JNM-ECZS 400M (JEOL Co., Tokyo, Japan) spectrometer. Experimental details are described for the determination of the oleate surface coverage (*vide infra*). For that purpose, Rutherford backscattering spectrometry (RBS) measurements were conducted on a Pelletron 5SDH2 (National Electrostatics Corp., Middleton, USA) with a He^{2+}-ion-beam source (2.274 MeV). The detector was placed at a backscattering angle of 160°. RBS samples were prepared by spin coating PbS toluene solutions (10 mg/mL) on Si wafers (2×2 cm) with a rate of 1500 rpm for 30 s, followed by storing under vacuum until the measurements were carried out. Scattering signals were collected until the charge integration had reached 10 μC.

6. Determination of the Pb/S and Cl/Pb ratios

RBS was used to measure the atomic Pb/S ratio (R) of GPC-2 and before-GPC (PR1) QDs. The measurements were carried out using a 2.274 MeV He^{2+}-ion beam. Based on the backscattered yield, R is determined by:

$$R_{Pb/S} = \frac{A_{Pb}}{A_S}\left(\frac{Z_S}{Z_{Pb}}\right)^2 \quad (1)$$

where A_{Pb} and A_S are the peak area of the Pb and S atoms, respectively, while Z_{Pb} and Z_S are the atomic number of Pb and S, respectively[2].

7. Determination of the ligand density from TGA

TGA was used to determine the ligand density of GPC-processed QDs. The weight ratio of the ligand OA and QD can be determined using the TGA curves. The number of atoms (N) in a single QD can be estimated from:

$$N = \frac{4\pi}{3}\left(\frac{d}{a}\right)^3 \quad (2)$$

where d is the QD diameter and a is the lattice constant of the PbS rocksalt structure[2]. The weight of a single QD can be calculated by N and the atomic Pb/S ratio (1.27) obtained from RBS. Based on the weight of the QD and the ligand OA, the ligand density (the number of the ligands per square nm) can be calculated.

8. Determination of the ligand density by NMR spectroscopy

The ligand density (nm^{-2}) was also determined by ^1H NMR spectroscopy and optical absorbance following a modified version of a reported procedure[3]. GPC samples with the known weight were dispersed in anhydrous toluene-d_8 (0.6 mL) with ferrocene (0.05 mg/mL). The molar concentration of the oleate ligands was determined via the internal reference according to:

$$[OA] = [FC] \times \frac{10}{I_{FC}} \times \frac{I_{OA}}{2} \quad (3)$$

where [OA] and [FC] are the molar concentration of the oleate ligands and ferrocene, respectively. I_{FC} and I_{OA} are the integral peak area of ferrocene and oleate ligands in the ^{1}H NMR spectra, respectively[3]. Due to the presence of free OA in the samples, the integral area of the ligand was calibrated by a Gaussian fit to subtract the peak of free OA (Figure S3).

The molar concentration of the QDs was determined by optical absorption of the QDs used in the NMR measurements according to the size-dependence of the molar extinction coefficient of QDs[2]. For that purpose, the NMR solutions were diluted with TCE, and the absorbance of these solutions was measured at 3.1 eV (400 nm). The molar concentration of the QDs (*C*) was estimated according to:

$$C = \frac{A_{3.1\ eV}}{0.0233(ld^3)} \quad (4)$$

where $A_{3.1\ \mathrm{eV}}$ is the absorbance at 3.1 eV, *l* is the path length (cm), and *d* is the QD diameter (nm). According to the molar concentration of the ligand OA and QD, the ligand density (the number of the ligands per square nm) can be calculated[3].

9. Preparation of liquid/air assembly films

Liquid/air assembly films were prepared according to a literature procedure[4]. A before-GPC toluene solution was carefully dropped onto the liquid plane of DMSO in a small container. TEM grids and quartz glass were used as substrates, and the self-assembled films were transferred on these. The self-assembled films on the substrates were immersed into methanol in order to remove excess DMSO and then dried *in vacuo* at room temperature.

Reference

1. M. C. Weidman, M. E. Beck, R. S. Hoffman, F. Prins and W. A. Tisdale, *ACS Nano*, 2014, **8**, 6363-6371.
2. I. Moreels, K. Lambert, D. Smeets, D. De Muynck, T. Nollet, J. C. Martins, F. Vanhaecke, A. Vantomme, C. Delerue, G. Allan and Z. Hens, *ACS Nano*, 2009, **3**, 3023-3030.
3. A. Abelson, C. Qian, T. Salk, Z. Luan, K. Fu, J. G. Zheng, J. L. Wardini and M. Law, *Nat. Mater.*, 2020, **19**, 49-55.
4. L. Liu, S. Z. Bisri, Y. Ishida, T. Aida and Y. Iwasa, *Nanoscale*, 2019, **11**, 20467-20474.

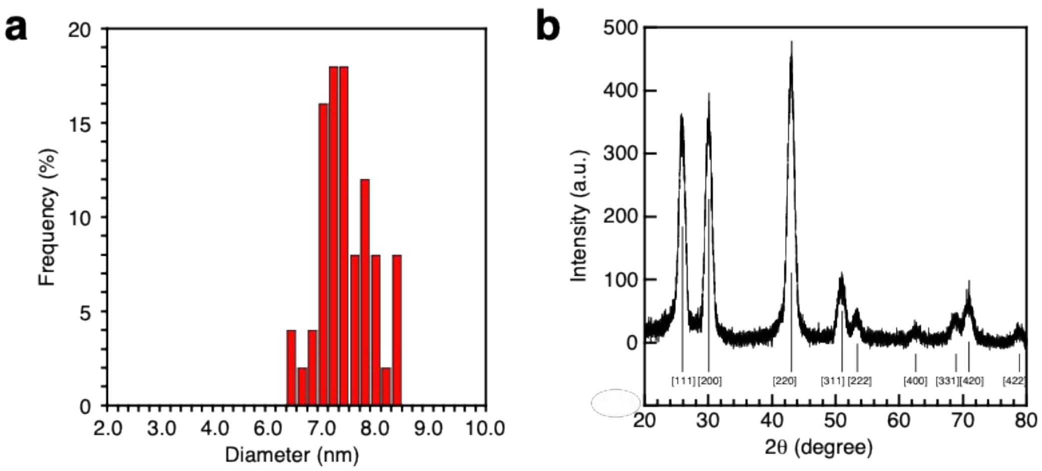

Figure S1. (a) Size distribution of before-GPC QDs. (b) XRD diffraction of before-GPC QDs.

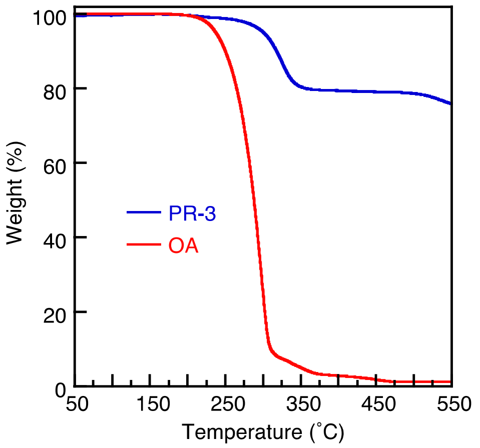

Figure S2. TGA curves of PbS QDs without unbound OA after three times PR purification (PR-3) and pure OA.

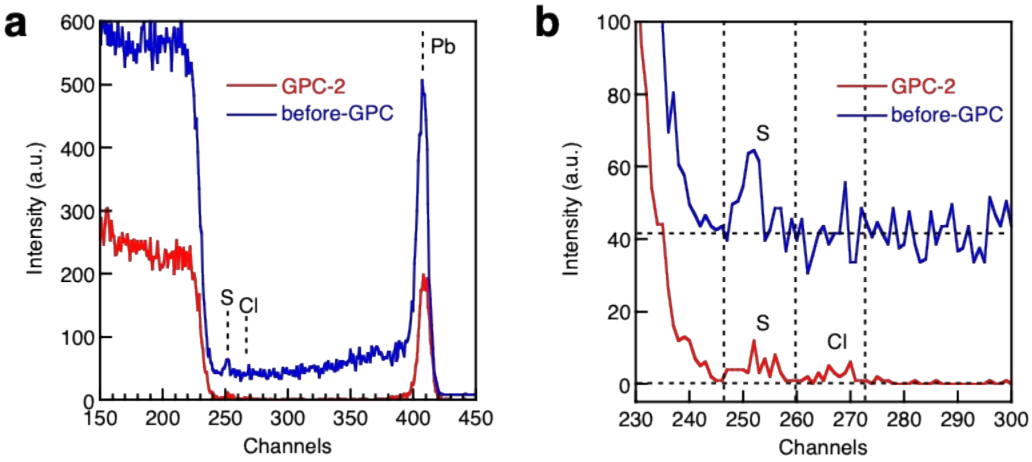

Figure S3. (a) Rutherford backscattering (RBS) spectra of GPC-2 and before-GPC (PR1) PbS QD films (150-450 channels). (b) Enlarged graph (230-300 channels).

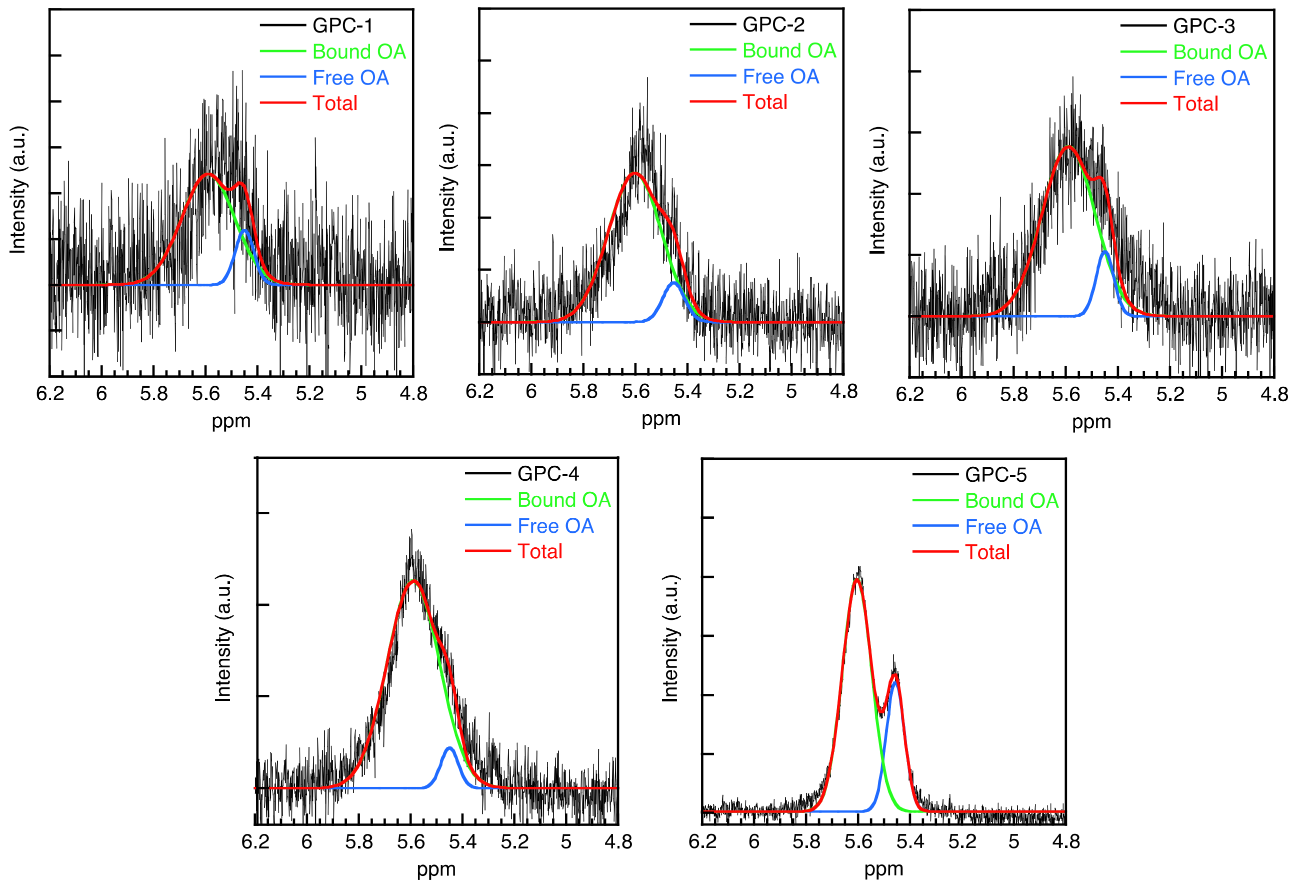

Figure S4. ^{1}H NMR spectra (area corresponding to the OA ligands) for GPC1-5 PbS QDs and their corresponding Gaussian peak separation of bound OA and free OA; black line: experimental; green line: bound OA; blue line: free OA; red line: total.

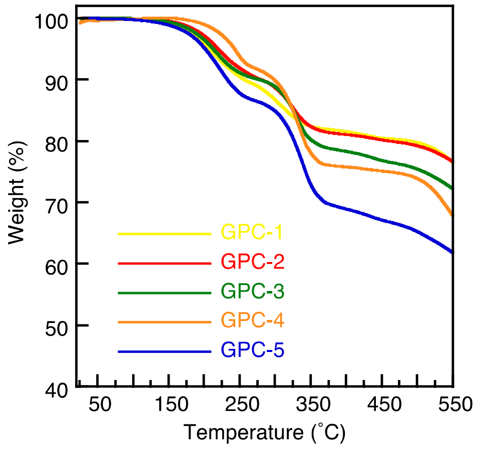

Figure S5. TGA curves of GPC1-5 PbS QDs using super anhydrous toluene as the eluent in the GPC process.

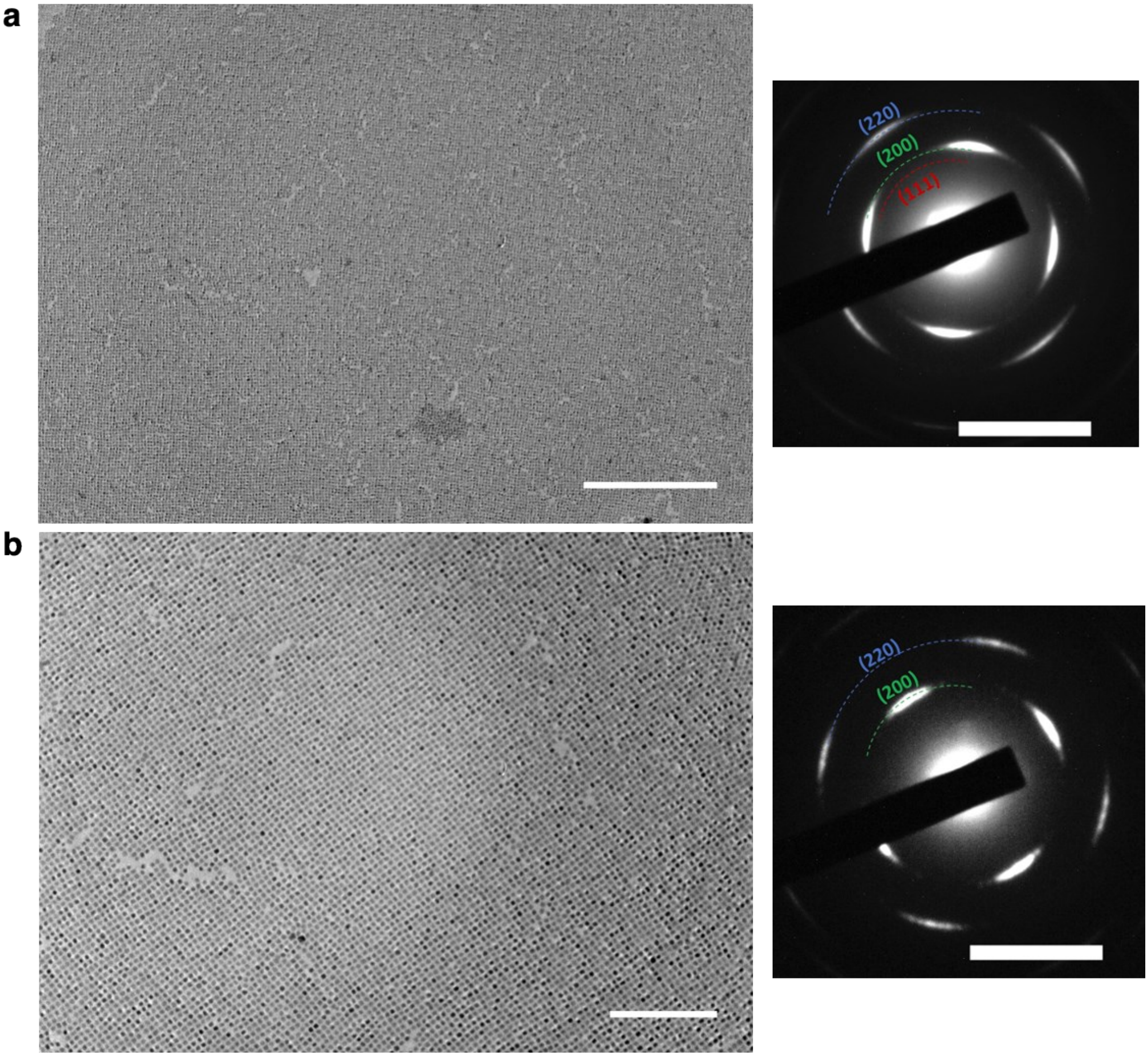

Figure S6. TEM images and SAED patterns of 2D self-assemblies for GPC-2 QDs; scale bars: (a) 500 nm (TEM) and 5 nm^{-1} (SAED). (b) 200 nm (TEM) and 5 nm^{-1} (SAED).

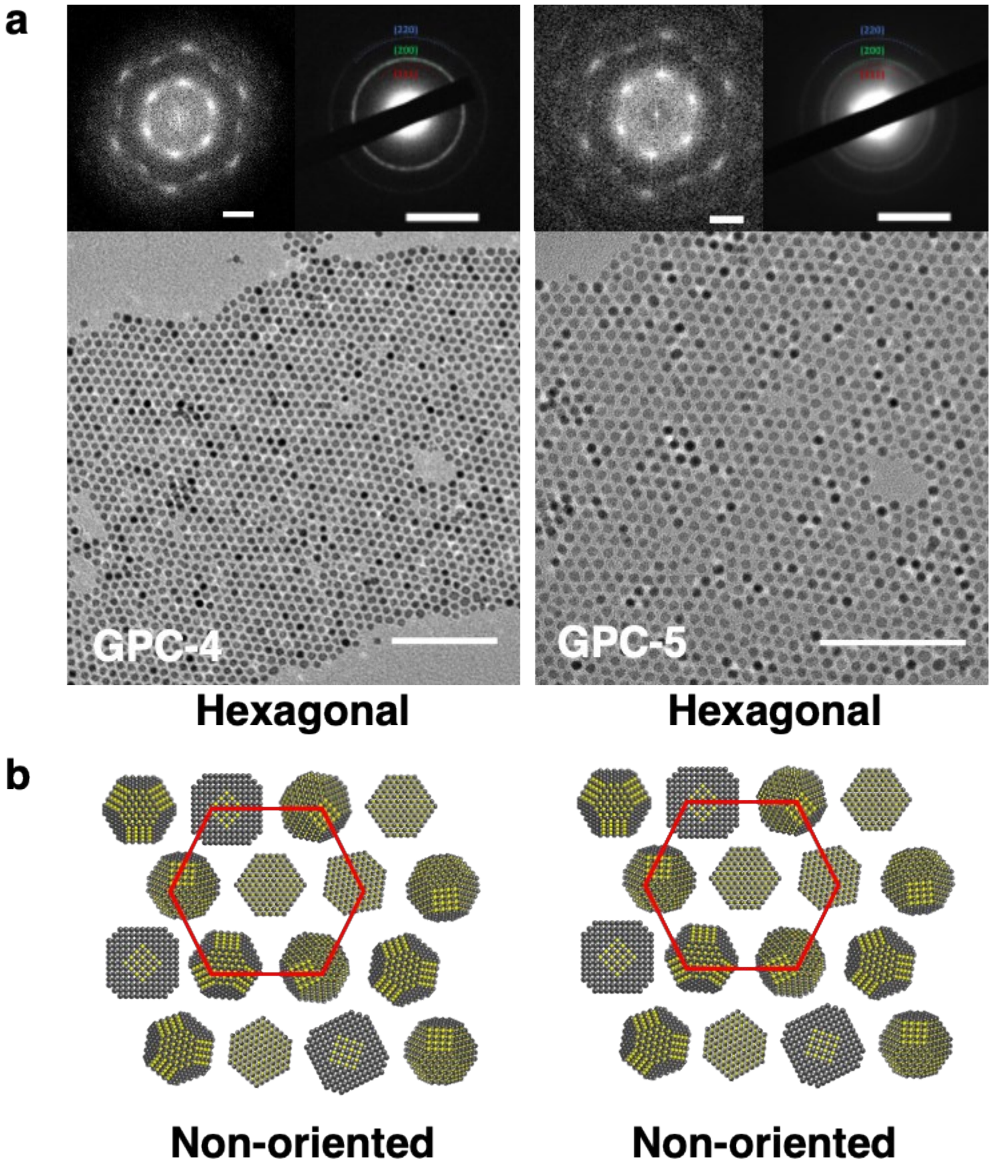

Figure S7. (a) TEM images of 2D self-assemblies for GPC-4 and GPC-5 QDs; inset: FFT and SAED patterns; scale bars: 100 nm (TEM), 0.1 nm^{-1} (FFT), and 5 nm^{-1} (SAED). (b) Schematic diagrams of the corresponding 2D self-assemblies.

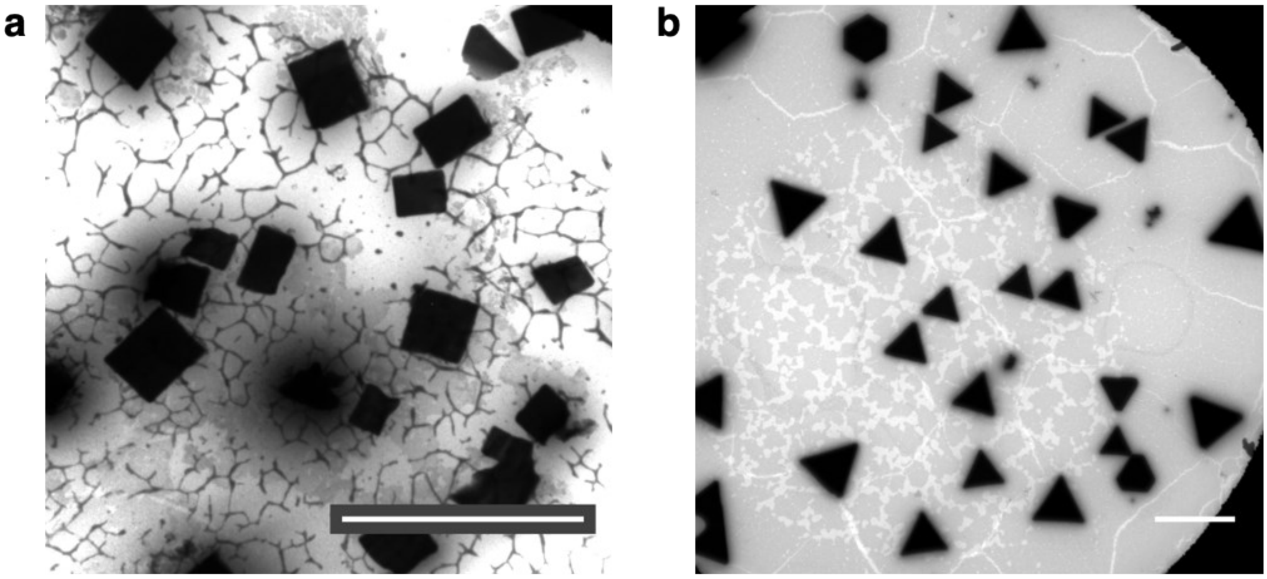

Figure S8. TEM images of 3D supercrystals for (a) GPC-2 and (b) GPC-5 QDs; scale bar: 10 µm.

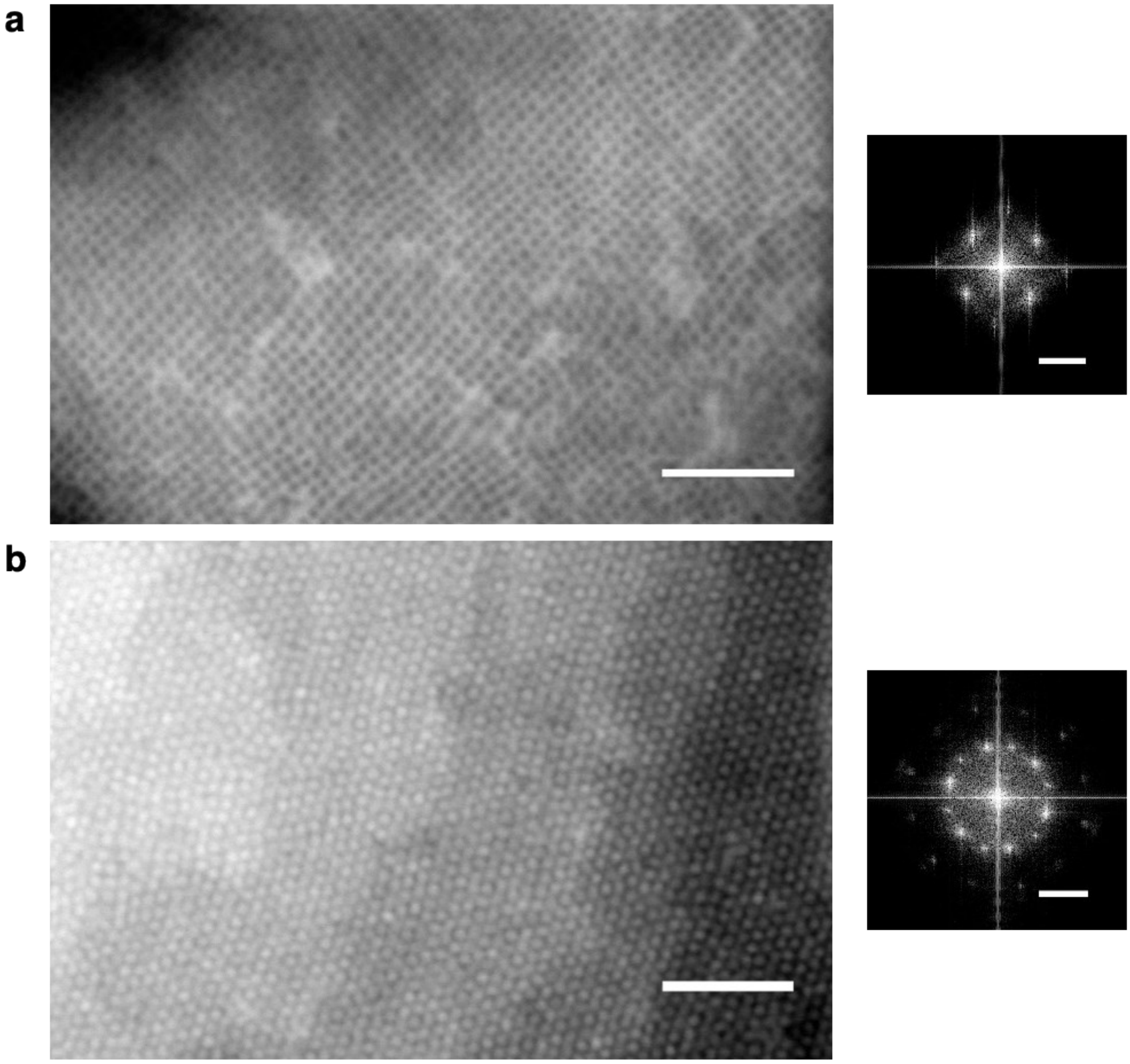

Figure S9. STEM images and FFT patterns of supercrystal surfaces for (a) GPC-2 and (b) GPC-5 QDs; scale bars: 100 nm (STEM) and 0.1 nm^{-1} (FFT).

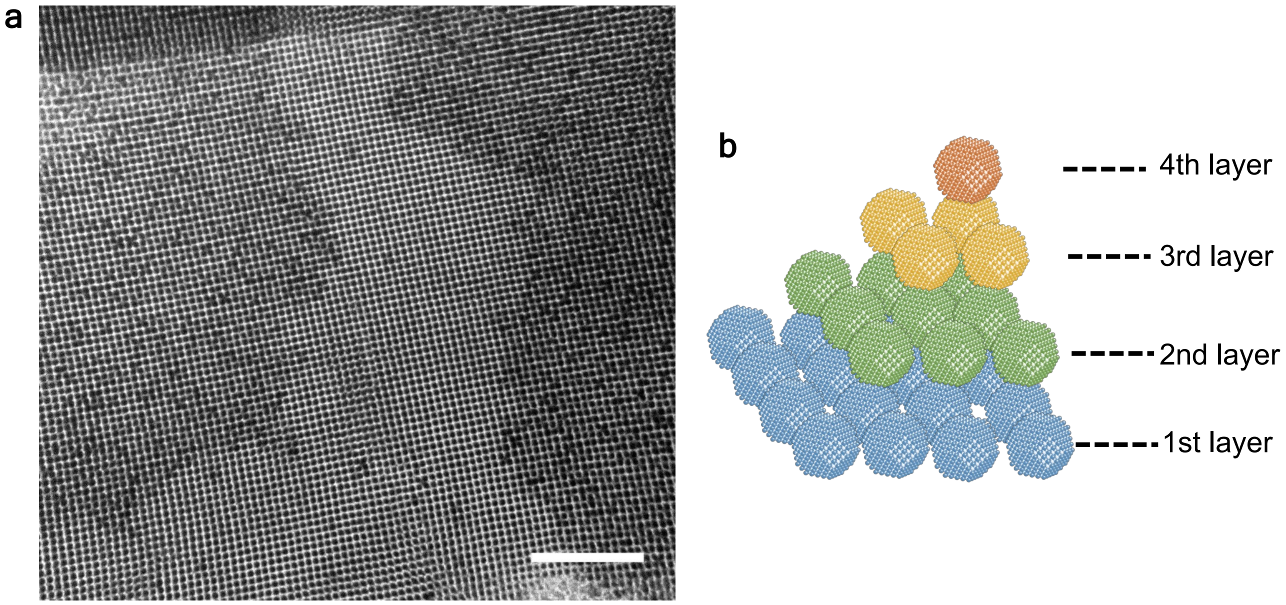

Figure S10. (a) TEM image of square multilayer superlattices for GPC-2 QDs. (a) Schematic model of the multilayer *sc* superlattice; scale bar: 100 nm.

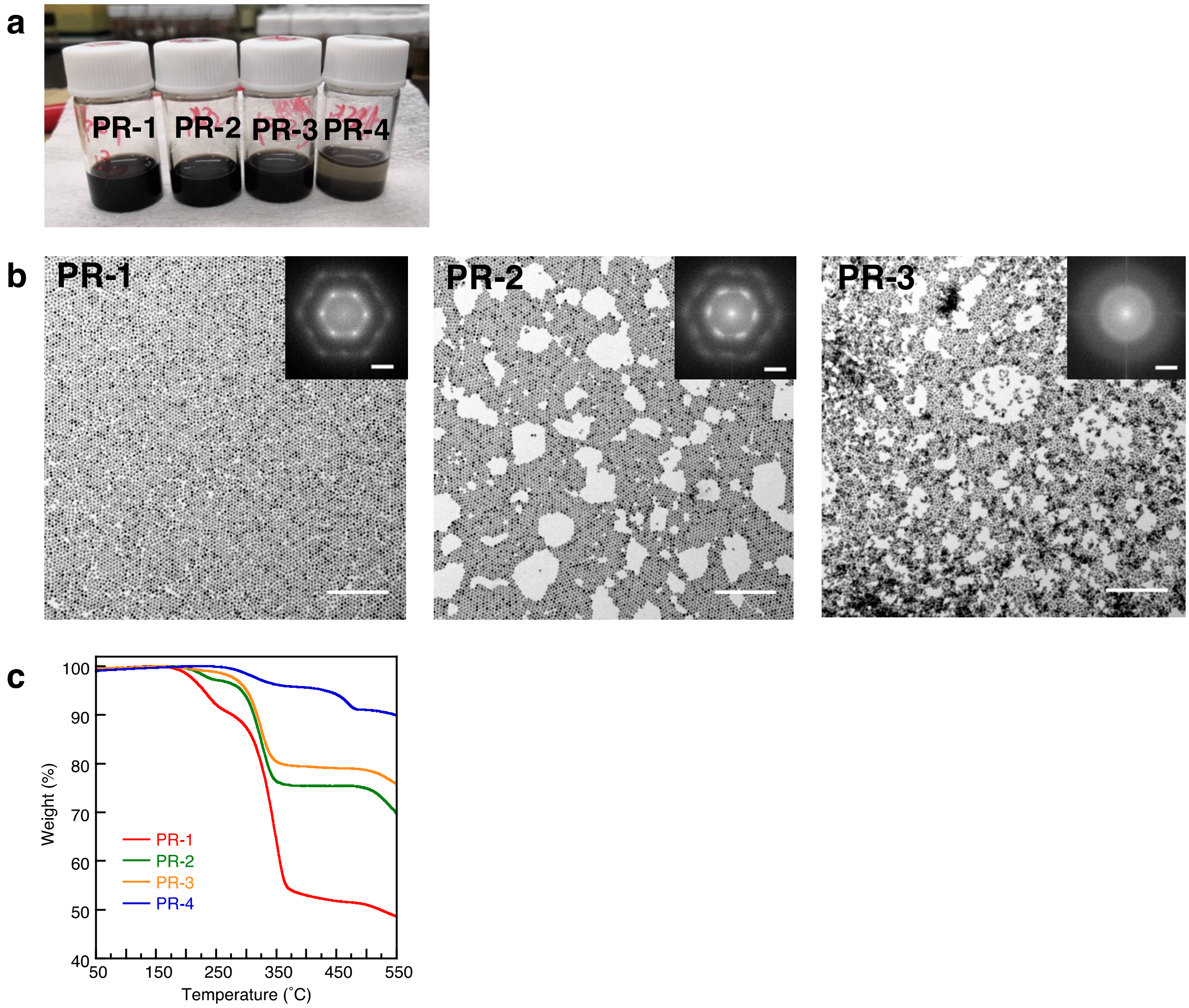

Figure S11. (a) Photograph of PbS QDs in dispersion after the PR processes (PR1-4). (b) TEM and FFT images of PR1-3 QDs; scale bars: 200 nm (TEM) and 0.1 nm^{-1} (FFT). (c) The TGA curves of PR 1-4 QDs.

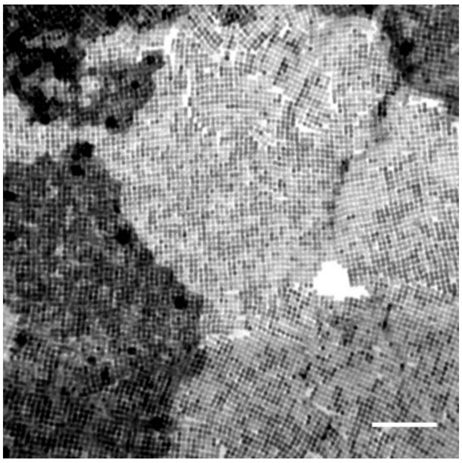

Figure S12. TEM image of the 2D assembly for before-GPC QDs obtained from the liquid/air interface method with DMSO; scale bar: 100 nm.

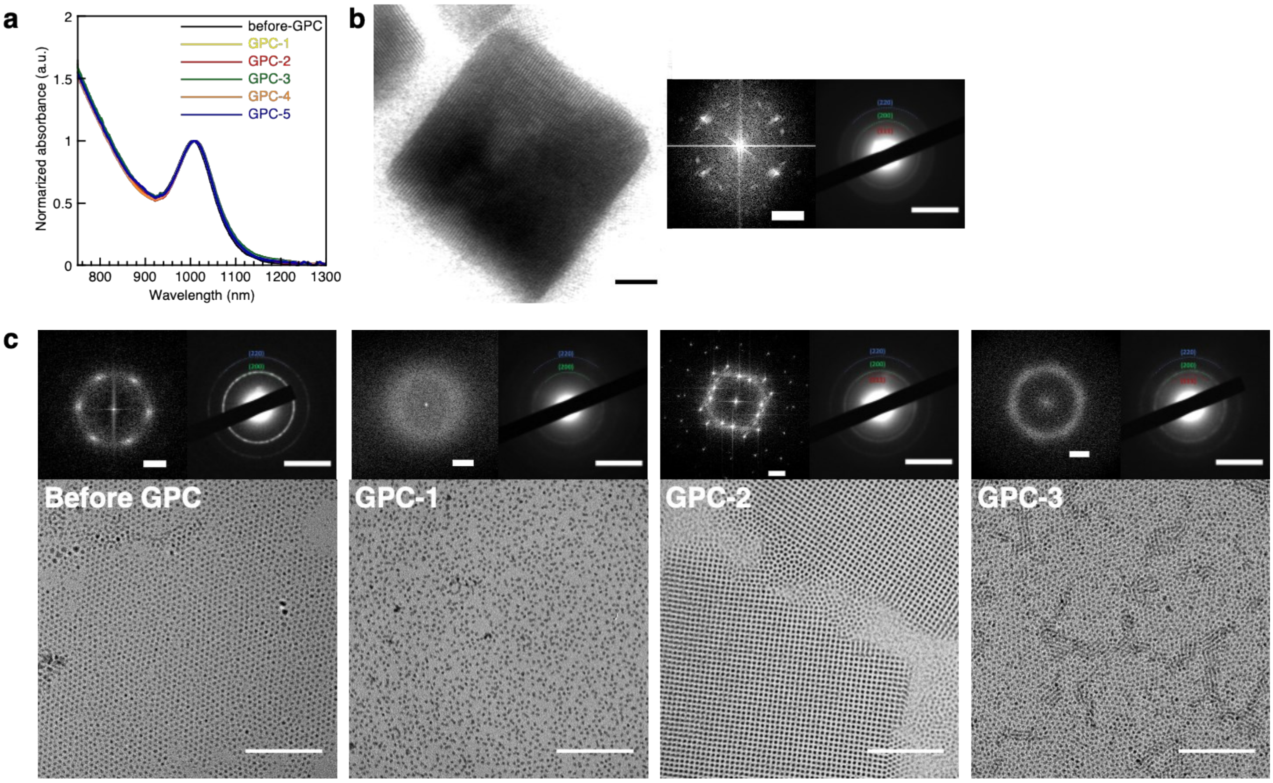

Figure S13. Small PbS QDs (average diameter: 4.3 nm): (a) Absorption spectra of before-GPC and GPC 1-3 QDs in tetrachloroethylene (TCE) solution. (b) TEM and FFT images of self-assembled supercrystals of GPC-2 QDs. (c) TEM images of the 2D self-assemblies of before-GPC, GPC-1, GPC-2, and GPC-3 QDs; inset: FFT; scale bars: 100 nm (TEM) and 0.1 nm^{-1} (FFT).
